# Barriers to care for mental health conditions in Canada

**DOI:** 10.1371/journal.pmen.0000065

**Published:** 2024-09-19

**Authors:** Monnica T. Williams, Muna Osman, Aidan Kaplan, Sonya C. Faber

**Affiliations:** 1 School of Psychology, University of Ottawa, Ottawa, Ontario, Canada; 2 Dept of Cellular and Molecular Medicine, University of Ottawa, Ottawa, Ontario, Canada; 3 School of Epidemiology, University of Ottawa, Ottawa, Ontario, Canada; University of Luxembourg: Universite du Luxembourg, LUXEMBOURG

## Abstract

There are growing concerns in Canada about access barriers to quality mental health care, which has worsened significantly by the COVID-19 pandemic and for some Canadians more than others. With a nationally representative sample of 1501 adults, surveyed by the Angus Reid Institute, this study examined the mental health conditions Canadians experience the most difficulties in accessing care. Among half of the respondents who sought mental health care, the majority encountered challenges in accessing help for posttraumatic stress disorder (PTSD) (34%) and depression (33%). When examining the data based only on those seeking care for specific conditions, attention deficit hyperactivity disorder (ADHD), obsessive-compulsive disorder (OCD), substance use disorders, and generalized anxiety disorder (GAD) emerged as those for which it was most difficult to find treatment. Indigenous and Black Canadians had significantly more difficulty finding care across several conditions. We discuss the implications of these findings, including the critical need to increase the supply and diversity of mental health providers across Canada. This study is one of the first to provide quantitative data on the perceived barriers in accessing mental health care, while exploring the role of race and ethnicity and other social identities.

## Introduction

### Mental health needs and care in Canada

Canada, like many countries around the globe, has ongoing challenges in the provision and availability of mental health services. Not only do Canadians lack adequate access to mental health care, the amount of care needed is actually increasing. Each year one in five Canadians require treatment for mental illness. About half of Canadians will be diagnosed with a mental illness by the age of 40 years [1]. In 2017, 5.3 million Canadians reported needing mental health services, half (3 million) had their needs fully met; from the remaining half, 1.2 million had their needs partially met, and 1.7 million had their needs entirely unmet [2]. The most commonly reported reasons for experiencing unmet or partially met mental health needs were: lack of time, lack of financial means, and a limited information or knowledge.

Canadian health care system is federally funded to provide necessary coverage of hospital and physician services, and this system is managed, organized, and delivered differently across individual provinces and territories. Existing health and social policy gaps span the range of mental health care including promotion, prevention resources, as well as intensive specialized services [3]. Additionally, longstanding gaps in health insurance coverage and employment-based health benefits creates inequities and contributes to growing rates of unmet mental health care needs suggesting this is a dominant reality for many Canadians [4]. Policy and funding gaps exacerbate a two-tier system of public and private access to care [4], and these gaps were intensified by the COVID-19 pandemic. Notably, almost 70% of Canadians experienced adverse effects from the pandemic, while by the end of 2020, 40% reported experiencing mental distress in the previous month [5] (Statistics Canada, 2021).

A recent scoping review identified a range of patient and clinician related challenges, which include system capacity, need for continued training, and complexity across different services, settings, and providers [6]. Issues related to the availability and complexity of mental health care was reported in most of the included studies, along with issues of capacity, education, training, fragmented services and limited resources [6]. To build on this literature, the purpose of this study is to examine the mental health conditions Canadians experience the most difficulties in accessing care and disparities in these experiences among individuals with diverse identities and backgrounds.

### Mental health conditions in Canada

The most concerning mental health conditions can be broadly divided into four areas: mood disorders, anxiety disorders, schizophrenia spectrum disorders, and substance use disorders. The prevalence of these conditions often varies by generation and cultural background. However, the lack of data collection by ethnic and racial groupings in Canada hinders our understanding of racial differences.

#### Anxiety disorders

In a lifetime, 4.6% or 2.5 million Canadians suffer from an anxiety disorder. A national survey in July 2020 found one in seven Canadians (13.6%) were at risk of clinically significant levels of generalized anxiety disorders [7] which was further exacerbated by the COVID pandemic. Although prevalence of anxiety disorders in women was higher than men (17.2% vs. 9.9%), a dose-response relationship with COVID-19 misinformation exposure was only observed among men [7], underscoring the need for improved public health information access.

A community survey on mental health in 2012 found that 2.6%, or about 1,000,000 Canadians over the age of 15 reported experiencing “symptoms consistent with generalized anxiety disorder” [8]. The 12-month prevalence of GAD was 3.2% in women and 2.0% in men [9].

#### Obsessive-compulsive disorder

A study of 25,097 Canadians found an obsessive compulsive disorder (OCD) diagnosis in 0.93% of the population [10]. Additionally, those with OCD were younger (M_age_ = 37.35), than the control group (M_age_ = 45.73) and were less likely to hold a job and had lower incomes than those without OCD [10]. These patterns highlight the impact of mental health experiences on socio-economic challenges.

#### Posttraumatic stress disorder

In 2021, Statistics Canada found that 8% of Canadians “met the criteria for probable PTSD”, whereas only 5% were diagnosed by a health professional, highlighting the lack of clinicians available to diagnose and treat individuals with this condition [11]. Women reported experiencing PTSD (10%) at rates almost twice as high as men (6%). Furthermore, individuals aged 18–24 years old reported having more PTSD symptoms (13%) than those 65+ years old (3%) and only 7% of these individuals identified as visible minorities [11]. Additionally, only half of those who suffer from PTSD (55%) sought external support, and of that, 82% “had trouble accessing the health care services they needed” [11, 12].

#### Depression & suicide

In a 2020 survey, 15% of Canadians screened positive for MDD, with more women diagnosed (18%) than men (13%). The highest proportion of MDD diagnosis were among those aged 18–34 years (23%) [5].

Untreated, depression can progress to suicidal ideation. Daily, 11 people in Canada take their own life resulting in about 4,000 deaths per year [13]. In 2018, suicide was the leading cause of death for children aged 10 to 14, and after accidents, it remained as the second leading cause of death for people aged 15 to 24 [13]. Indigenous people, particularly youth, have significantly increased rates of suicide. First Nations youth between 15 to 24 years of age experience suicide rates about six-fold higher than other Canadians. Among Inuit youth, suicide rates are about 24 times higher than the national average [14].

#### Schizophrenia spectrum disorders

Canadians already living with serious mental illnesses, including psychotic disorders, were profoundly impacted by the COVID-19 pandemic. These vulnerable Canadians were not only at higher risk for contracting COVID-19, the social distancing protocols and disruptions in routine services created a higher risk for poor mental health outcomes [15]. Psychotic disorders including schizophrenia have been determined to affect up to 4% of the population [16]. This means that more than 1.5 million Canadians are directly affected. The onset of schizophrenia, which occurs in early adulthood or late adolescence, is particularly tragic as it negatively affects the life experiences of young Canadians during a time when they are embarking on an independent life [17]. Globally it is among the top 10 causes of disability-adjusted life-years [16], and the costs in Canada of schizophrenia per year has been estimated to reach up to $10 billion Canadian dollars, demonstrating the urgent need for better care in this area [17].

#### Substance use disorders

The prevalence of substance use disorder in Canada is at 1.78% representing over 650,000 Canadians [18], and each day, on average, 20 Canadians perish from the use of illicit substances [19]. Mortality caused by the use of alcohol, opiates and other substances can be grouped together as “deaths of despair” and are on the rise in Canada. Although opioid-related deaths have been highest in British Columbia, Alberta, Yukon, and the Northwest Territories, the crisis has touched all regions of Canada [19, 20]. In all, the economic cost of substance use in Canada per year is a staggering $40 billion, which includes criminal justice, lost productivity and healthcare costs [19, 21].

#### Eating disorders

The most common eating disorders include anorexia nervosa (AN), bulimia nervosa (BN), and binge-eating disorder. The lifetime prevalence of eating disorders is notably higher (8.4%) for women than men (2.2%), and this trend extends to the prevalence for anorexia nervosa at 1.4% for women and 0.2% for men as well as bulimia nervosa 1.9% for women and 0.6% for men, and finally, for binge eating disorder where women have a prevalence of 2.8% compared to men (1.0%) [22, 23]. Individuals with eating disorders may be undertreated due to avoidance behaviors as the stigma and self-stigma around eating disorders has been reported to obstruct help-seeking behavior [24]. Additionally, the COVID-19 pandemic has severed social connections exacerbating the negative impacts of eating disorders [24].

#### Attention deficit hyperactivity disorder

Attention Deficit Hyperactivity Disorder (ADHD) symptoms commonly arise in children between the ages of 3 and 5, remain throughout adolescence in 75% of cases, and persist in 50% throughout adulthood [25]. One study found that from 1999 to 2012, ADHD had risen in all provinces for both youth, aged between 1–17 and young adults aged between 18–24 [26]. The prevalence of ADHD in adults is 2.5%, and in youth, between 4%-7%, with a three-fold higher likelihood in boys of ADHD development than in girls [25].

Caregivers of children with ADHD were adversely affected due to restrictions of the pandemic, as they were unable to access services they required (therapeutic, educational or medical), which resulted in an increase and worsening of symptoms [26–28].

#### Dementia

Dementia, characterized by memory loss, judgement and reasoning problems, behavioral alterations, and mood and communication disturbances, is one of the more expensive mental health disorders costing Canada about 8.3 billion in 2011 [29]. The prevalence of dementia increases with age, with an overall prevalence of 2.0% for the general population [30, 31], however the national data found a prevalence rate of 7.1% for dementia in individuals over 65 years old [32], with women (8.3%) experiencing higher levels of dementia than men (5.6%) [33]. Pandemic restrictions, including enforced social isolation, had severely negative impacts on people living with dementia [34, 35].

#### Systemic racism

Systemic racism is prevalent in Canada and exacerbates all mental health conditions. When compared to White Canadians, Canadians of color receive substandard mental health care, and face more barriers when seeking support [36]. Discrimination also negatively affects the mental health of racialized Canadians and causes racial trauma which, although it has long existed was only recently formally recognized by psychologists (i.e., changes in DSM) [36, 37]. Only recently has the burden of racism in Canada been elevated to become a central theme within the national conversation [15, 37].

### Purpose of this paper

The main objective of this paper is to investigate disparities in access to mental health care among marginalized and racialized Canadians. It focuses on analyzing differences in access to care for specific mental health conditions, considering factors such as gender, location, age, and importantly, ethnoracial background. This study builds upon previous survey research that has identified barriers to accessing mental health services in Canada [38].

## Materials and methods

### Ethics statement

The Angus Reid Institute, a national research organization founded in 2014, collected the data for this report from a national survey of Canadian adults between February 22–24, 2022 in both English and French. Members of the Reid Institute online forum were provided with written informed consent, information on the purpose of the survey, compensation, steps on how to withdraw, and the protection of private information in the terms of service and privacy policy upon registration. Anonymous unidentifiable data were provided to the authors, and therefore, they did not require ethics approval from the University of Ottawa Research Ethics Board (REB) for this investigation.

### Participants

This study includes a representative Canadian sample of 1501 individuals. Regional representation included the Atlantic region (7%), Alberta (11%), British Columbia (13%), Ontario (38%), Quebec (24%) and Saskatchewan/Manitoba (7%). For gender, 48% identified as male and 52% identified as female. For education levels, participants had lower than or equal to high school education (37%), partial/some postsecondary/college education (33%), and a university degree or more (30%). For household income: 28% earned less than $50k, 35% earned between $50k and $100k, and 27% earned more than $100k. For ethnoracial identity, the sample included: 18 identifying as Middle Eastern/West Asian, 22 as Black, 1179 as White, 41 as East Asian (including Chinese, Taiwanese, Hongkonger, and other East Asian), 38 as South Asian, 24 as Latin American, 92 as Indigenous Canadian and 87 as “other” ethnicities (which included Filipino, multiethnic, and prefer not to answer).

### Survey questions

Participants were asked, “To the best of your knowledge, what types of mental health problems are most difficult finding help for? Please select all that apply.” The 14 options were: attention deficit hyperactivity disorder (ADHD), generalised anxiety disorder (GAD), obsessive compulsive disorder (OCD), posttraumatic stress disorder (PTSD), depression, bipolar disorder, schizophrenia, Alzheimer’s/dementia, eating disorders (e.g. anorexia, bulimia, binge eating, etc.), developmental disorders, alcohol/drug abuse, other/please specify, or none of the above.

### Data analyses

A series of analytical tests were implemented to compare responses across the conditions most associated with difficulties and the demographic variables, including contingencies tables, chi-square independence tests, post-hoc pairwise z-tests. The demographic variables analyzed include language, household income levels, provinces of residence, education levels, ethnic and racial identity, age groups and gender. independence tests were used to study variation in the frequencies across levels of demographic variables. Post-hoc pairwise z-tests examined whether the observed frequencies were significantly different across demographic variables.

Independent samples t-tests and one-way Analysis of Variance (ANOVA) were used to investigate mean level differences across each value of the demographic variables. Posteriori tests in ANOVA were used to investigate significant pairwise comparisons. The data was balanced and weighted for age, education, gender, and the provinces to be representative of the Canadian population.

## Results

### To the best of your knowledge, what types of mental health problems are most difficult to find help for?

Most respondents identified posttraumatic stress disorder (PTSD; 33%) and depression (33%) as the most difficult conditions to access care. Other conditions reported included generalized anxiety disorder (GAD; 27%), bipolar disorder (27%), schizophrenia (25%), eating disorders (23%), ADHD (21%), Alzheimer’s/dementia (21%), OCD (20%), developmental disorders (20%), alcohol/drug abuse (20%), and other/please specify (2%). From the few who selected *other*, participants mentioned personality disorders (Borderline, Antisocial, Narcissistic), and addictions (e.g., gambling, online social media) among other conditions.

**Fig 1:**
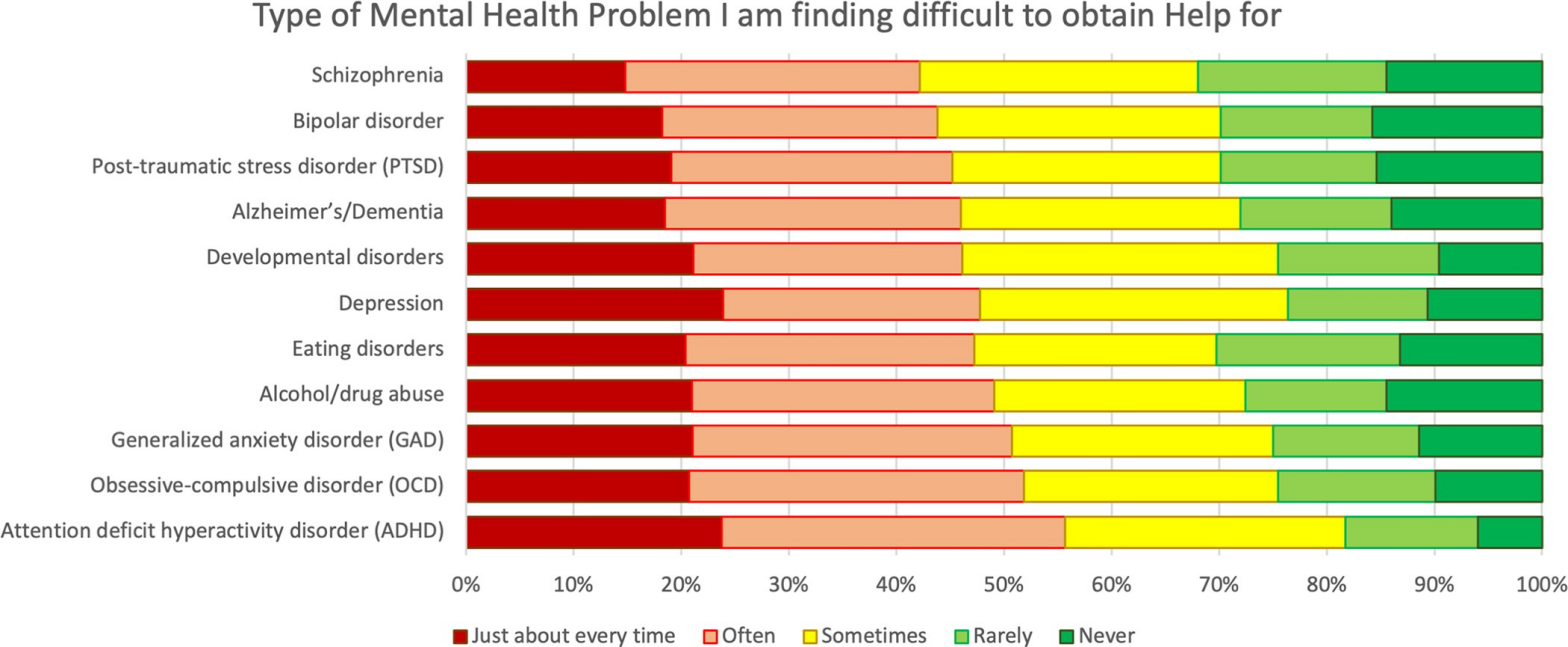
Levels of difficulty by indication and barriers to mental health care. Conditions listed include only those who reported seeking help for the condition listed in the y-axis.

Table 1 and Fig 1 show the national prevalence of each condition based on the literature [2, 12, 18, 22, 23, 39, 40], followed by the percentage of those who sought help for the condition, and those who had difficulty finding help, based on our analysis. The prevalence rates are lower than rates of seeking care because individuals might seek care for other people, including family. Among those who sought out care, ADHD was deemed most difficult to find care for, followed by OCD, SUD and GAD. To explore these patterns, we examined differences across seven demographic variables.

**Table 1:** Difficulty finding help and prevalence rates.

Disorder	Prevalence in Canada	Ever sought help for condition (regardless of barriers)	Had difficulty* finding help for the condition
Alzheimer’s/Dementia	2.0%	13.3%	46.0%
Developmental disorders	1.1%	13.9%	46.2%
Substance (alcohol/drug) abuse	2.2%/1.78%	14.3%	49.1%
Obsessive-compulsive disorder (OCD)	2.5%	14.1%	51.9%
Attention deficit hyperactivity disorder (ADHD)	2.1%	14.6%	55.7%
Eating disorders	0.42%	15.7%	47.2%
Schizophrenia	0.42%	17.5%	42.2%
Generalized anxiety disorder (GAD)	2.57%	18.7%	50.7%
Bipolar disorder	1.51%	19.0%	43.9%
Post-traumatic stress disorder (PTSD)	5.0%	21.7%	45.2%
Depression	4.72%	22.6%	47.8%

Note: Out of those reporting that they ever experienced a barrier to finding mental health care for a specific condition. Includes those who searched for help for the listed disorder, responding with having difficulty “often” or “just about every time.”

### Race/Ethnicity

**Fig 2:**
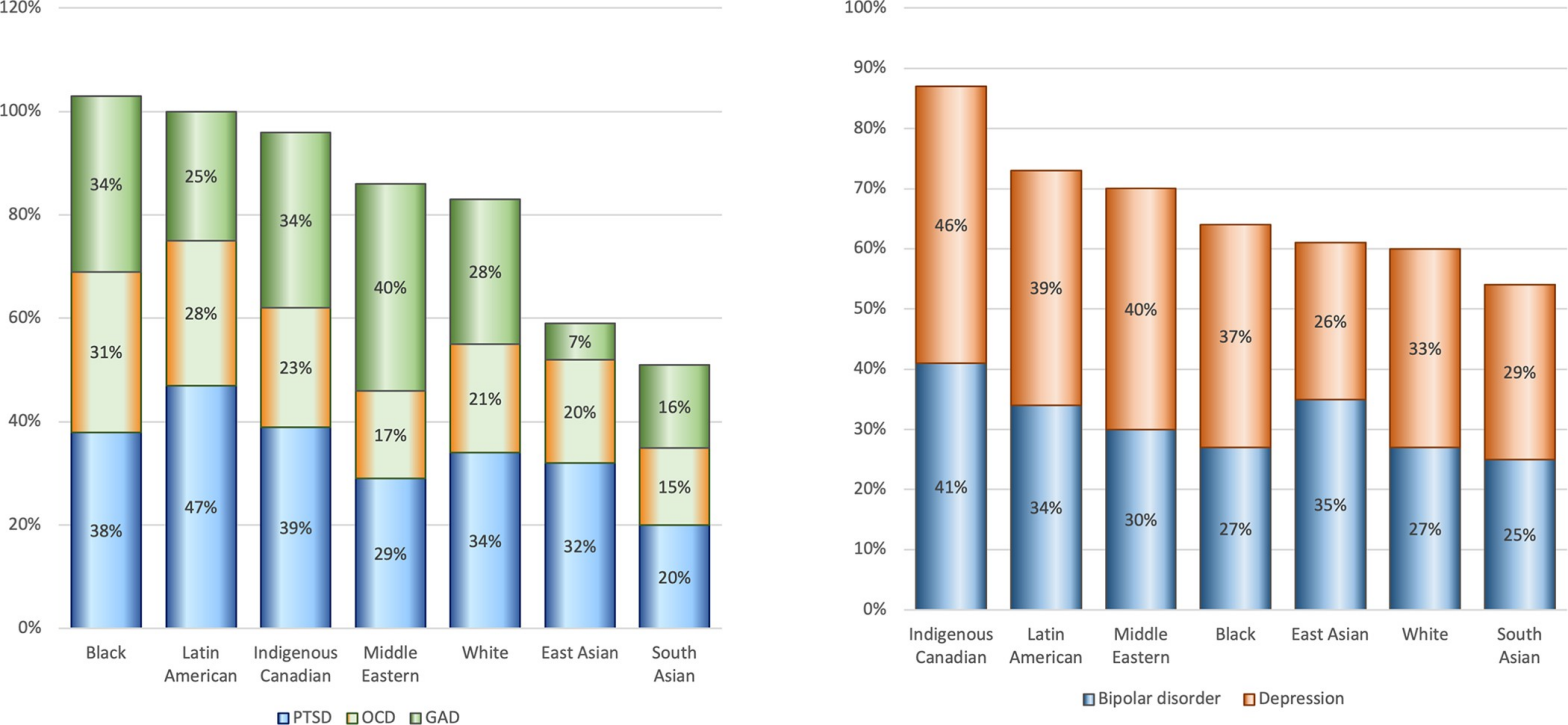
Difficulty accessing care for mental health conditions by ethnoracial group. Difficulties accessing care for mood-related disorders (left) and anxiety-related disorders (right).

Fig 2 shows the proportion of individuals having difficulties accessing care for mental health conditions by ethnoracial group. Ethnicity was significantly related to mean differences in the conditions most associated with difficulties (F(6,1407) = 2.668, p = 0.014). Specifically, Indigenous people reported significantly more difficulties associated with general anxiety disorder (GAD) and other conditions compared to other groups. Specifically, Indigenous people reported ADHD (30%), depression (46%), and bipolar disorder (38%) more than White respondents (20%, 32%, and 27%, respectively). Indigenous people, Black, and White respondents reported PTSD (38%, 28%, and 34%) more than South Asians (21%). Similarly, Indigenous people, Black, and White respondents reported GAD (36%, 24%, 27%) more than East Asian (7%). Black Canadians reported Alzheimer’s/Dementia (33%) more than South Asian (16%) and Middle Eastern/West Asian (11%). Access difficulties due to schizophrenia were not different across ethnicity.

### Province of residence

Provinces were significantly related to mean differences in the conditions most associated with difficulties, specifically for developmental disorders (F(5,1495) = 4.513, p<0.001) and ADHD (F(5,1495) = 2.506, p = 0.029). Given the highest proportions of respondents (approximately 56%) who sought out care and reported difficulties were from Ontario and Atlantic Canada [38], these regions report difficulties for more conditions. Ontario (23%) and Atlantic Canada (29%) reported ADHD as being more related to difficulties, compared to Alberta (16%) and Quebec (17%). Also, Ontario (35%) and Atlantic (41%) reported PTSD as more related to difficulties than Quebec (28%). In Ontario, schizophrenia (28%) was associated with more difficulties than Quebec (22%). Depression was reported as more related to difficulties in Ontario (34%), Quebec (35%), and Atlantic Canada (40%), than British Columbia (25%).

### Gender

Gender differences were found for several conditions, including ADHD (t(1498) = -2.321, p = 0.020), GAD (t(1498) = -4.126, p<0.001), OCD (t(1498) = -3.186, p<0.001), Schizophrenia (t(1498) = -4.207, p<0.001), eating disorders (t(1498) = -4.949, p<0.001), developmental disorders (t(1498) = -3.496, p<0.001), and alcohol/drug abuse (t(1498) = -5.763, p<0.001). Women reported significantly more conditions than men, including ADHD (23% and 18%), GAD (32% and 22%), OCD (23% and 17%), schizophrenia (29% and 20%), SUD (26% and 14%) as well as eating (28% and 17%) and developmental (23% and 16%) disorders.

### Age

Age differences were found for ADHD (F(2, 1498) = 8.976, p<0.001), depression (F(2, 1498) = 3.324, p = 0.036), eating disorders (F(2, 1498) = 8.891, p<0.001), and developmental disorders (F(2, 1498) = 8.314, p<0.001). Young adults (aged 18 to 34) and middle-aged (aged 35–54) were more likely than older adults (55 and older) to identify ADHD (24%, 24%, and 15% respectively) as most difficult condition. In general, young adults reported more conditions than older adults, specifically, OCD (23% and 18%), schizophrenia (29% and 22%), as well as eating (30% and 19%) and developmental (25% and 15%) disorders.

### Education level

Education was only related to differences in the conditions most associated with difficulties, specifically, GAD (F(2, 1498) = 1.604, p = 0.017) and Depression (F(2, 1498) = 12.171, p<0.001). Canadians with less than or at least high school or some college education were more likely than those with university education to report most difficulties for GAD (29%, 29%, and 22%) and depression (38%, 35%, and 24%).

### Household income

Differences based on income was found for PTSD (F(2, 1498) = 3.882, p = 0.021), depression (F(2, 1498) = 3.482, p = 0.031), bipolar disorder (F(2, 1498) = 5.174, p = 0.006), and developmental disorders (F(2, 1498) = 3.134, p = 0.044). Canadians who made less than $50k or $50K-100k reported more conditions associated with difficulties, than those making over 100k; conditions reported included PTSD (37%, 35%, and 29%), bipolar disorder (32%, 28%, and 23%), and schizophrenia (27%, 26%, and 21%) as being related to the most difficulties in accessing care. Depression was reported more by Canadians with less than $50K household income than those making over $100K (37% and 29%).

### Language

Differences in language was related PTSD (t(1499) = 2.822, p = 0.002) and alcohol/drug abuse (t(1499) = 2.739, p = 0.006) such that more English-speaking Canadians than French-speaking reported PTSD (35% and 26%) and alcohol/drug abuse (21% and 14%) as more difficult to access care.

## Discussion

### Mental health conditions and access to care

All Canadians do not have equal access to mental health care. Respondents reported varying level of access for different conditions, however, they indicated experiencing difficulty “just about every time” and “often” for all surveyed mental health conditions between 42% and 56% of the time. Consistent with research, women sought out more care and experienced more difficulties in general than men [38].

Although most Canadians were seeking services for depression and PTSD, when adjusted by proportion, ADHD was the most condition to find help for, with over half of the sample experiencing difficulties almost always or often. ADHD is often identified during the school years through a school psychologists [41, 42], however, given shortages of these types of professionals, children are medicated rather than receiving therapy [43].

Closely behind ADHD, were anxiety-related conditions, including OCD, SUD, and GAD. Psychotherapy is more effective and is, in the long-term, more enduring than treatment with medications alone, especially for these conditions [44]. OCD is difficult to find treatment for, as medication rarely provides full relief, and few therapists are trained in CBT treatments, such as exposure and ritual prevention [40]. Correspondingly, the need for therapy was found the most likely to remain unmet (34%) while the need for medication was most likely to be met (85%) [45]. Therapy is more cost-effective and leads to fewer relapses of anxiety and depression than medication use alone [45, 46].

### Racial barriers to mental health

Indigenous people experienced the highest barriers to accessing care as compared to other groups, with 30 to 40% reporting treatment barriers for ADHD, depression, and bipolar disorder at rates significantly higher than White respondents. Barriers to treatment for anxiety disorders, PTSD, and GAD were also reported highest by Indigenous people, at over a third, followed by Black and White Canadians. Asian ethnicities (South and East Asian) reported the least barriers.

Data shows race-based differences in the prevalence of mental health conditions [20, 47, 48]. Indigenous youth have higher suicide rates than other young Canadians, while the prevalence of mood disorders among immigrants and Black people differs from White Canadians [14, 49, 50]. Some racialized communities have higher rates and needs for anxiety treatment than White communities, while Black communities in both the US and Canada have lower rates of substance use than White communities [20, 49, 51]. In contrast, rates of depression among Black Canadians rose to nearly six times the prevalence of the Canadian population [47]. These differences inform the rates of help seeking observed for various conditions in this study.

### Unequal distribution of resources and access to care

Disparities in mental health care in Canada is rooted in the unequal distribution of resources and access to care, such that certain populations are disproportionately experience barriers and are therefore less likely to receive appropriate treatment. Gaps in the supply, diversity, and cultural competence of providers are key factors contributing to barriers. This gap in turn perpetuates the power imbalance, as those with access to services are more likely to receive adequate care, while those who do not are left without the necessary care. Addressing this power imbalance is crucial to ensure all Canadians have access to the care they need and deserve.

### Recommendations

This study demonstrates the growing need for more mental health professionals, services, and resources for all Canadians. For example, there is a need for more licensed clinical psychologists (18,000 in Canada, whereas there are 102,000 in the US), and to address the well-known administrative and professional bottlenecks in the Canadian education system for clinical psychologists [52–54]. To address this issue, the US has free-standing professional schools graduating more than half of the US-based clinical psychologists. Yet, geographic disparities and discrimination impact race-based shortages in professionals for racialized communities in both countries [54, 55]. There is some progress now, in Canada, to accredit free-standing professional degrees and university-based PsyD programs, and Canadian Psychological Association’s (CPA) most recent accreditation standards released in only 2023 now accommodate this [43, 56].

The limited availability of services in languages other than English hampers access to services for diverse immigrants [57]. Although resource-intensive interpretation services can be helpful [58], a better alternative is to train and accredit more multilingual therapists locally or better recognize credentials from other countries. Unfortunately, health professionals in Canada cannot be licensed in languages other than English and French (e.g., [59]).

In Canada, the only systematic governmentally mandated collection of race-based data is for Indigenous people. Special direct action is warranted for Indigenous people [43], as our own investigation could only identify 5 Indigenous psychology faculty out of over 1,200 in the province of Ontario [60], and many experience barriers, such as being required to leave their communities to pursue clinical education and training [61, 62]. This study further highlights difficulties in access experienced by Indigenous people and racialized communities.

Graduate programs should improve training for specific conditions that are particularly difficult to find specialists such as ADHD. Training programs should highlight access deficits for specific conditions and encourage subspecialties to meet these demands.

Further, Canada should embrace breakthrough therapies such as psychedelics [63]. Psychedelics show promise for the treatment of PTSD, depression and anxiety, and other conditions [64], and a new Psychedelics educational programming at the University of Ottawa (e.g., an MA program) provides educational opportunities for these innovative approaches [65]. Finally, continuing education in culturally-informed approaches and anti-racist proficiency is essential [66]. Skill gaps in professionals contributes to difficulties in access to competent mental health services for racialized people.

### Limitations & future directions

There are several limitations to consider for this study. The online format and inclusion criteria could limit the participation of non-English or French speaking individuals or those with limited internet access. Also, geographically, no territories were included in this study, where marked difficulties in accessing mental health care might be particularly important. Future surveys should ensure the full inclusion of Indigenous persons on reserves, rural populations, and institutionalised persons, as the mental health needs of these groups are critical as well.

## Conclusion

This study is a quantitative synthesis of the conditions many Canadians are experiencing barriers in accessing care. Access to care for many mental health conditions is unacceptably difficult, and even more so for Indigenous people, immigrant, racialized communities. Given the lack of sufficient and timely race-based data, there is no policy remedy for these trends. Equitable solutions must consider race-based differences in the prevalence and treatment of conditions.
